# Human T cells loaded with superparamagnetic iron oxide nanoparticles retain antigen-specific TCR functionality

**DOI:** 10.3389/fimmu.2023.1223695

**Published:** 2023-08-17

**Authors:** Felix Pfister, Jan Dörrie, Niels Schaft, Vera Buchele, Harald Unterweger, Lucas R. Carnell, Patrick Schreier, Rene Stein, Markéta Kubánková, Jochen Guck, Holger Hackstein, Christoph Alexiou, Christina Janko

**Affiliations:** ^1^ Department of Otorhinolaryngology, Head and Neck Surgery, Section of Experimental Oncology and Nanomedicine (SEON), Else Kröner-Fresenius-Stiftung Professorship, University Hospital Erlangen, Erlangen, Germany; ^2^ Department of Dermatology, University Hospital Erlangen, Erlangen, Germany; ^3^ Department of Transfusion Medicine and Hemostaseology, University Hospital Erlangen, Erlangen, Germany; ^4^ Organic Chemisty Laboratory, Department of Biochemistry, University of Bayreuth, Bayreuth, Germany; ^5^ Faculty of Applied Natural Sciences and Health, Hochschule Coburg, Coburg, Germany; ^6^ Max-Planck-Institute for the Science of Light & Max-Planck-Zentrum für Physik und Medizin, Erlangen, Germany; ^7^ Department of Physics, Friedrich-Alexander-Universität Erlangen-Nürnberg (FAU), Erlangen, Germany

**Keywords:** Adoptive T cell therapy, superparamagnetic iron oxide nanoparticles (SPIONs), antigen-specific T cell response, targeted immune therapy, cell deformability

## Abstract

**Background:**

Immunotherapy of cancer is an emerging field with the potential to improve long-term survival. Thus far, adoptive transfer of tumor-specific T cells represents an effective treatment option for tumors of the hematological system such as lymphoma, leukemia or myeloma. However, in solid tumors, treatment efficacy is low owing to the immunosuppressive microenvironment, on-target/off-tumor toxicity, limited extravasation out of the blood vessel, or ineffective trafficking of T cells into the tumor region. Superparamagnetic iron oxide nanoparticles (SPIONs) can make cells magnetically controllable for the site-specific enrichment.

**Methods:**

In this study, we investigated the influence of SPION-loading on primary human T cells for the magnetically targeted adoptive T cell therapy. For this, we analyzed cellular mechanics and the T cell response after stimulation via an exogenous T cell receptor (TCR) specific for the melanoma antigen MelanA or the endogenous TCR specific for the cytomegalovirus antigen pp65 and compared them to T cells that had not received SPIONs.

**Results:**

SPION-loading of human T cells showed no influence on cellular mechanics, therefore retaining their ability to deform to external pressure. Additionally, SPION-loading did not impair the T cell proliferation, expression of activation markers, cytokine secretion, and tumor cell killing after antigen-specific activation mediated by the TCR.

**Conclusion:**

In summary, we demonstrated that SPION-loading of T cells did not affect cellular mechanics or the functionality of the endogenous or an exogenous TCR, which allows future approaches using SPIONs for the magnetically enrichment of T cells in solid tumors.

## Graphical Abstract

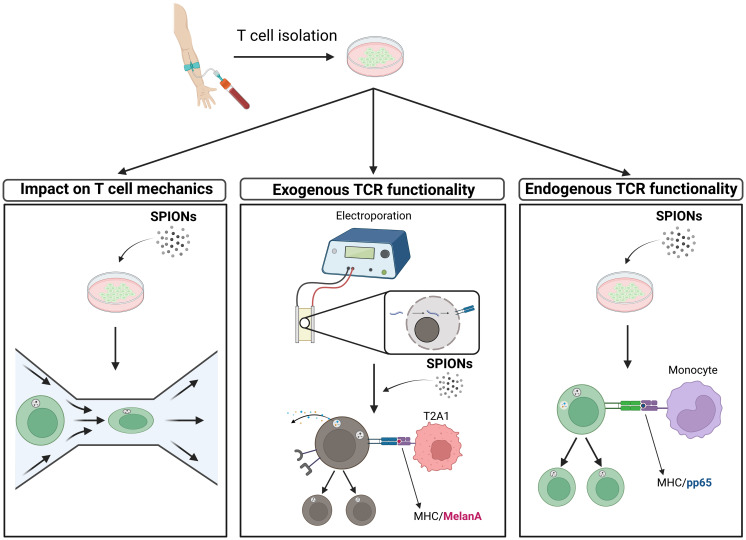

## 1 Introduction

Cancers represent a very high burden of disease in the population, with nearly 2 million new cases and more than 600,000 deaths in the U.S. alone (1). Adoptive T cell therapy is a cell-based type of immunotherapy as a treatment option for advanced cancers (2). This therapy utilizes T cells acquired from blood or tumor tissue, which are expanded and optionally genetically modified to increase the anti-tumor effect (3). These modifications include the expression of an exogenous T cell receptor (TCR) or a chimeric antigen receptor (CAR), which enables the detection of tumor antigens, resulting in an anti-tumor immune response. To date, only CAR-T cell therapies targeting hematological cancers are approved. However, adoptive T cell therapies for solid tumors are still in the experimental or early clinical stages (4). In contrast to cancers of the blood, solid tumors form a dense extracellular matrix composed of collagen and fibronectin, which limits the non-proteolytic T cell infiltration (5). Additionally, the tumor microenvironment restrains T cell metabolism and effector functions owing to its hypoxic and nutrient-depleted nature (6, 7). Solid tumors are also able to induce immunosuppression directly via the blocking of checkpoint receptors or indirectly through recruited cells like tumor-associated macrophages or T regulatory cells (8, 9). These limitations result in either insufficient T cell infiltration or low efficacy. Moreover, adoptive T cell therapy is limited by severe systemic side effects, such as off-target toxicity and cytokine release syndrome (10, 11). These challenges have been approached previously by e.g. thorough research for tumor-specific neoantigens or directly by clinical intervention via immunosuppressive agents (3, 12). Nevertheless, lack of a suitable neo-antigen and immunosuppression may dampen the clinical effectiveness. Thus, there is a need to increase tumor-infiltration of T cells, while decreasing the distribution of applied anti-cancer specific T cells in healthy tissues to minimize potential on-target/off-tumor effects.

The magnetic controllability of superparamagnetic iron oxide nanoparticles (SPIONs) has been used previously as a method to enrich therapeutic drugs in a desired region, referred to as “Magnetic Drug Targeting” (13). SPIONs, loaded with a chemotherapeutic agent, were successfully enriched in the desired area via an external magnetic field, resulting in enhanced anti-tumor activity (14, 15). Furthermore, it has been shown that these particles can be detected via X-ray microtomography or magnetic resonance imaging, thus acting as a contrast agent (16–18). Additionally, magnetic particles have also played a role in the tissue engineering of the vascular system (19). SPIONs have also been used to magnetically control immune cells and enrich them in the target area (20–27). In the context of adoptive T cell therapy, the main application area of SPIONs is the visualization and tracking of therapeutic T cells *in vivo (* 28, 29 *).* Prior studies using citrate-coated SPIONs showed that T cells remained viable and functional after polyclonal stimulation (22, 23). Nanoparticles were stably bound on the surface of T cells as well as taken up intracellularly, allowing the magnetic enrichment in a dynamic flow system (23, 30). However, it was unclear, if SPION-loading has an impact on T cell mechanics. To address this, we performed within this outlined study a real-time deformability cytometry (RT-DC), which allows label-free analysis of the mechanical properties of T cells (31). Additionally, we analyzed the influence of SPIONs on the ability of human T cells to recognize and react to antigens with either their endogenous TCR or an exogenously introduced TCR, via the expression of activation markers or cytokines and proliferation.

## 2 Materials and methods

### 2.1 Materials

Cell culture plates were obtained from TPP (Trasadingen, Switzerland). The Muse^®^ Count & Viability Assay Kit was purchased from Merck (Darmstadt, Germany). Annexin A5 (AxV)-fluorescein isothiocyanate (FITC), AxV-Allophycocyanin (APC), Hoechst 33342 (Hoe), Gibco RPMI medium 1640, Opti-MEM™ without phenol red, GlutaMAX supplement, Penicillin-Streptomycin (PS) solution 5000 U/mL, 50 mM 2-mercapthoethanol, 1000x Brefeldin A-solution, PE-Cy7 anti-human CD107a, eFluor™ 450 anti-human Interleukin (IL)-2, fixable viability dye eFluor™ 450, SYTOX™ Blue Nucleic Acid Stain, and fixable viability dye eFluor™ 780 were bought from Thermo Fisher Scientific (Waltham, MA, USA). Propium iodide (PI) and phosphate buffered saline (PBS) were acquired from Sigma-Aldrich (Taufkirchen, Germany). Biochrom (Berlin, Germany) provided fetal calf serum (FCS) and Amphotericin B. Human CD3 Fab-TACS Gravity Kit was purchased from IBA (Goettingen, Germany), and 65% nitric acid and formaldehyde solution from Carl Roth (Karlsruhe, Germany). Miltenyi Biotec (Bergisch Gladbach) provided the Inside Stain Kit, APC-Vio770^®^ anti-human IFNγ, PE anti-human TNFα, and FcR blocking reagent. 1000x Monensin, Alexa Fluor^®^ 700 anti-human CD3, PerCP/Cy5.5 anti-human CD4, APC/Cyanine7 anti-human CD4, Pacific Blue™ anti-human CD8a, APC anti-human CD8a, PerCP/Cyanine5.5 anti-human CD8a, Brilliant Violet 605™ anti-human CD14, PE anti-human CD25, FITC anti-human CD69, Alexa Fluor^®^ 647 anti-human IFNγ, CFSE cell division tracker kit, ELISA MAX™ Standard Set Human IFNγ, ELISA MAX™ Standard Set Human TNFα, ELISA MAX™ Standard Set Human IL-2, LEGENDplex™ Human CD8/NK Panel, Human Pan Monocyte Isolation Kit, and Zombie Aqua™ Fixable Viability Kit were purchased from BioLegend (San Diego, CA, USA). Lymphocyte separation media was purchased from anprotec (Bruckberg, Germany). Recombinant human IL-7 was obtained from ImmunoTools (Bösel, Germany), and Histopaque1077 and saponin from Sigma-Aldrich (St. Louis, MO, USA). PepMix™ HCMVA (pp65) and PepMix™ Human (MOG) were purchased from JPT Peptide Technologies (Berlin, Germany). BIOWEST (Nuaillé, France) supplied 1 M HEPES buffer, whereas gentamycin (10 mg/mL) and human serum from male AB plasma was obtained from Bio & Sell (Fürth, Germany). S-Monovette^®^ Citrate 3.2% and 21G Safety-Multifly^®^ needles were purchased from Sarstedt (Nümbrecht, Germany). Ibidi GmbH (Gräfelfing, Germany) supplied tissue culture treated µ-slide I Luer. All deionized H_2_O (dH_2_O) was produced in-house using a Merck Milli-Q Direct water purification system (Darmstadt, Germany). Beckmann Coulter (Brea, CA, USA) provided the Gallios Flow Cytometer, the CytoFLEX S flow cytometer and the data analysis software Kaluza (Version 2.1). Additionally, the data was also analyzed using FlowJo (Version 10.8.1.) purchased from Tree Star Inc (Ashland, OR, US). The SpectraMax iD3 Plate reader was purchased from Molecular Devices (San José, USA). The Gene Pulser Xcell electroporator was obtained from Bio-Rad Laboratories (Hercules, CA, USA) and 4 mm electroporation cuvette from Peqlab (Erlangen, Germany) or BioLabProducts GmbH (Bebensee, Germany). Zeiss (Oberkochen, Germany) provided the Axio Observer Z1 fluorescence microscope and imaging software ZEN (Version 3.6).

### 2.2 SPION synthesis and physiochemical characterization

SPIONs were synthesized as described previously using an adjusted protocol by Elbialy et al. (23, 32) The nanoparticles were sterilized by filtration through a 0.2 µm syringe filter (Sartorius, Goettingen, Germany). The hydrodynamic size, iron concentration, magnetic susceptibility, and zeta potential of the SPIONs were then characterized as described by Mühlberger et al. and Boosz et al. (21, 23) The iron content was investigated at a dilution of 1:25 in dH_2_O, dissolved in 65% nitric acid, using atomic emission spectroscopy with an Agilent 4200 MP-AES (Agilent Technologies, Santa Clara, CA, USA) with an iron solution of 1000 mg Fe/L as an external standard (Bernd Kraft, Duisburg, Germany). Measurements were performed in triplicates at a wavelength of 371.993 nm, which were then averaged. SPIONs were diluted with sterile dH_2_O to the intended concentration for all experiments.

### 2.3 Isolation of T cells from human whole blood

Peripheral venous human whole blood (anticoagulated with tri-sodium citrate monovettes) for T cell isolation was obtained from healthy volunteers after informed consent (approved by the Ethics Committee of the Friedrich-Alexander-Universität Erlangen-Nürnberg; reference number 257_14 B) using a 21G Safety-Multifly^®^ needle. Alternatively, T cells were isolated directly from Leukoreduction system chambers (LRSC) (approved by the ethics committee of the Friedrich-Alexander-Universität Erlangen-Nürnberg; reference number 60_21B and 346_18B). CD3+ T cells were isolated using the CD3 Fab-TACS™ Gravity Kit, according to the manufacturer’s protocol for freshly drawn blood. T cell numbers were counted in a MUSE cell Analyzer using the MUSE^®^ Count and Viability assay kit (Burlington, MA, USA). The purity of T cell was determined using flow cytometry.

### 2.4 Cultivation and SPION-loading of human T cells

T cells were cultured in a humidified 5% CO_2_ atmosphere at 37°C in RPMI 1640 medium supplemented with 10% heat-inactivated FCS, 2% PS, 2 mM L-glutamine and 1% amphotericin B. T cell media used for experiments containing electroporated T cells was supplemented with an additional 10 ng/mL IL-7.

T cells were seeded at a concentration of 1 × 10^6^ cells/mL and loaded with 80 µg Fe/mL SPIONs overnight. A control group was established receiving only dH_2_O without SPIONs. The loading time for electroporated T cells with SPIONs was 4 h.

### 2.5 Influence of electroporation on SPION uptake

Jurkat T cells were cultured in a humidified 5% CO_2_ atmosphere at 37°C in RPMI1640, supplemented with 10% v/v FCS and 0.5% v/v PS. Before electroporation, cells were washed with RPMI 1640 medium and then with Opti-MEM™ (both room temperature). Cells were then resuspended at a maximal concentration of 2 × 10^7^ cells/mL in Opti-MEM™ and transferred into a 4 mm cuvette. Electroporation was performed with two square-wave pulses of 500 V/cm for 2 ms at 0.1 s intervals as described in (33), and immediately transferred into fresh medium. The controls group did not receive electroporation. Afterwards, Jurkat cells were loaded with either 40 or 80 µg Fe/mL SPIONs or only dH_2_O as a control and were incubated after loading for either 4 h or 24 h. Viability was investigated by flow cytometry and iron concentration was analyzed by AES, as described in 2.2. To investigate magnetical enrichment, Jurkat cells were electroporated as described above and loaded with 80 µg Fe/mL for 4 h, followed by staining with 20 µg/mL Hoechst 33342. The cells were then diluted to a concentration of 5 × 10^5^ cells/mL, of which 100 µL was added to a µ-slide. A magnetic field was introduced by the addition of a disc-shaped neodymium magnet (450 mT). The cells were then immediately analyzed using a Zeiss Axio Observer Z1 fluorescence microscope (Oberkochen, Germany), with the focal point being placed 5 mm from the magnet, and with pictures being taken every 15 s for 10 min. The images were analyzed using the ImageJ2 Plugin TrackMate (34, 35).

### 2.6 Real-time deformability cytometry of T cells

Before RT-DC analysis, measurement buffer containing 0.6% (m/v) methyl cellulose dissolved in PBS was prepared as described by Kubankova et al. (36) T cells were loaded with various SPION concentrations as described in Section 2.4, and then diluted in measurement buffer (room temperature) at a concentration of 1 × 10^7^ cells/mL. Measurements were performed using a commercial RT-DC instrument (AcCellerator, Zellmechanik Dresden GmbH) and a 20 x 20 µm microfluid chip made of polydimethylsiloxane at a temperature of 23°C as described in (36). Two gates were applied, namely for a minimal cellular cross-sectional area of 15 µm^2^ and 1.0 – 1.05 for the area ratio. Images of T cells were analyzed using the Shape-Out 2 software (37). Deformation and Young’s modulus of the cells were calculated as previously described (36).

### 2.7 Cultivation of T2A1 cells and MelanA peptide-loading

The TAP-deficient TxB cell hybrid and HLA-A2-positive cell line T2A1 (kindly provided by Prof. Georg Fey, Chair of Genetics, Erlangen, Germany) was cultured in RPMI 1640 supplemented with 10% heat-inactivated FCS, 2% PS, 2 mM L-glutamine, 2 mM HEPES buffer, 4 µg/mL gentamicin, and 2 mM 2-mercapthoethanol. T2A1 cells received 5 µg/mL of the HLA-A2-binding MelanA_26–35_ analogue ELAGIGILTV peptide (MelanA) for 2 h. As a control, no peptide was added to T2A1 cells. Afterwards, cells were washed to remove excess peptide and prepared for co-incubation with T cells.

### 2.8 Electroporation of T cells with MelanA-specific TCR mRNA

RNA synthesis and generation of MelanA-specific T cells via electroporation were performed as described before (38, 39). In short, T cells were washed with RPMI 1640 medium and then with Opti-MEM™ (both room temperature). Cells were then resuspended at a maximal concentration of 2 × 10^7^ cells/mL in Opti-MEM™ and transferred into a 4 mm cuvette. mRNA encoding the α- and β-chain of the MelanA-specific TCR (150 µg/mL mRNA each) was added to the cells. Electroporation with no mRNA was used as a negative control. The T cells were then pulsed with a square-wave pulse of 1250 V/cm for 5 ms and immediately transferred to fresh T cell medium. Afterwards, the cells were loaded with SPIONs as described in 2.4 for 4 h.

### 2.9 Analysis of antigen recognition and response of the exogenous TCR of SPION-loaded T cells

After electroporation and SPION-loading, 2 × 10^5^ T cells were seeded in a 96-well plate at a density of 1 × 10^6^ T cells/mL. For proliferation analysis, T cells were stained with CFSE as previously described (22). To detect intracellular cytokines, 0.1% v/v Brefeldin A solution was added to the T cells. To each condition 4 × 10^4^ T2A1 cells with or without MelanA were added and co-incubated over night for at least 12 h. At the next day, the supernatant of cells that were not treated with Brefeldin A was collected and frozen at -80 °C until the further use. Analysis of proliferation was performed by flow cytometry.

Expression of the activation markers was investigated by staining for CD4, CD8, CD25, and CD69, after which the expression was measured by flow cytometry. Intracellular cytokine content was analyzed after staining for CD4 and CD8. The cells were then fixed and permeabilized using the Inside Stain Kit according to the manufacturer’s instructions or fixed with 4% paraformaldehyde for 10 min at room temperature and then permeabilized with 0.5% saponin. The cells were then stained for IFNγ, TNFα, and IL-2 and analyzed by flow cytometry. Viability was controlled by staining the cells with a fixable viability dye prior to fixation. Degranulation was investigated by the addition of 1% v/v anti-human CD107a directly at the beginning of the co-incubation. After 1 h, 0.1% v/v monensin was added to the cells to reduce the degradation of fluorochrome conjugates due to the acidic environment of the lysosomes (40). At the next day, the cells were also stained for CD4 and CD8 and analyzed by flow cytometry. All samples were analyzed using a Gallios flow cytometer. Doublets, cell debris, and dead cells were excluded via forward and sideward scatter, as well as fixable viability dye eFluor™ 450 or fixable viability dye eFluor™ 780 staining. Activation markers or cytokines expressing CD8+ T cells were identified by gating on dead^-^CD8+ T cells.

The frozen supernatant was thawed and then immediately used to determine cytokine concentration. Cytokine concentrations of IFNγ, TNFα, and IL-2 in the supernatant were measured by ELISA, while additional cytokines were measured via a bead-based multiplex assay. Both procedures were performed in accordance to the manufacturer’s instructions.

### 2.10 Analysis of T cell-mediated cytotoxicity

Antigen-specific cytotoxicity of MelanA-specific T cells was investigated via flow cytometry or a ^51^chromium-release assay. T cells were electroporated as described in 2.8 and loaded with SPIONs for 4 h as shown in 2.4. Afterwards, the cells were washed and then co-incubated at a concentration of 2 × 10^5^ T cells for 16 h with 4 × 10^4^ T2A1 cells, which had been loaded with MelanA, as described in 2.7, and stained with 10 µM CFSE. The cells were then washed and stained with 1 µM Sytox for 20 min at 4°C and analyzed by flow cytometry afterwards. Doublets and cell debris were excluded via forward and sideward scatter, viable T2A1 cells were identified via CFSE+ Sytox-. For the chromium-release assay, T cells were kept in T cell medium in the incubator overnight. At the next day, T2A1 cells were labeled with 200 µCi of Na_2_^51^CrO^4^/10^6^ (PerkinElmer, Waltham, MA, USA) for 1 h and then with 5 µg/mL MelanA peptide for 1 h. Subsequently, T2A1 cells were washed, plated in a 96-well plate at 1000 cells/well, and co-cultured with T cells at various effector to target cells ratios (as indicated). After 4 h, supernatant was collected and chromium-content was measured with the Wallac 1450 MicroBeta plus Scintillation Counter (Wallac, Turku, Finnland). Percentage of cytolysis was calculated with the following equation:


$$100\%\;\times \frac{measured\;release-background\;release}{maximum\;release-\;background\;release}.$$

### 2.11 Analysis of antigen recognition and response of the endogenous TCR of SPION-loaded T cells

Peripheral blood mononuclear cells (PBMCs) were isolated from LRSC of cytomegalovirus (CMV) seropositive donors after written informed consent (approved by the ethics committee of the Friedrich-Alexander-Universität Erlangen-Nürnberg; reference number 346_18B). For that, LRSC content was mixed 1:1 with PBS prior to density centrifugation using lymphocyte separation media with a density of 1.077 g/mL. Pan monocytes were magnetically enriched from PBMCs by negative selection using the Human Pan Monocyte Isolation Kit, according to the manufacturer’s instructions. For the intracellular IFNγ analysis, 8 × 10^4^ monocytes and for the T cell proliferation assay, 4 × 10^4^ monocytes were seeded in a 96-well cell culture plate in 150 µL cell culture medium (RPMI-1640 supplemented with 100 U/mL penicillin, 100 µg/mL streptomycin and 10% human male AB serum). The cells were then incubated overnight until the T cells were added.

T cells were isolated, as described in 2.3, from the same LRSC as the monocytes and then loaded with SPIONs overnight as described in 2.4. At the next day, T cells were washed to remove excess SPIONs and counted. For the cytokine analysis, 3.2 in 50 µL cell culture medium were added to the monocytes. For the T cell proliferation assay, T cells were stained with 2.5 µM CFSE according to the manufacturer’s protocol as described above and then 1.6 × 10^5^ T cells were added to the monocytes in 50 µL cell culture medium. Co-cultured monocytes and T cells were stimulated with either 1 nmol/mL CMV-derived peptide pp65 or 1 nmol/mL myelin oligodendrocyte glycoprotein (MOG) peptide. No peptide was used as the negative control.

For intracellular IFNγ staining, 0.1% v/v Brefeldin A solution (BioLegend, San Diego, CA, USA) was added to the cell culture 1 h after stimulation. The cells were harvested and analyzed by flow cytometry after an additional 10 h of culture. To identify dead cells, the cells were stained with the Zombie Aqua Fixable Viability Kit in accordance to the manufacturer’s protocol. For extracellular antigen staining, cells were treated with FcR blocking reagent and afterwards incubated with anti-CD14, anti-CD3, anti-CD4, anti-CD8 for 20 min at 4°C in staining buffer consisting of PBS and 2% FCS. Afterwards, cells were fixed by incubation with PBS containing 2% formaldehyde for 5 min at room temperature. Cells were permeabilized by washing with FACS buffer containing 0.05% Saponin. Intracellular IFNγ was stained with anti-IFNγ in staining buffer containing 0.5% Saponin for 30 min at 4°C. Afterwards, the cells were analyzed by flow cytometry. For analysis of T cell proliferation, cells were harvested and analyzed by flow cytometry on day 5 after stimulation. Extracellular antigens were stained as described above for the IFNγ staining. To identify dead cells, 1 µM SYTOX Blue Dead Cell Stain was added 5 min before analyzing the samples via flow cytometry.

All samples were analyzed with a CytoFLEX S. Doublets, cell debris, and dead cells were excluded via forward and sideward scatter, as well as Zombie Aqua or SYTOX Blue staining, respectively. Auto-fluorescent cells were excluded via a dump channel. IFNγ+ CD8+ T cells were identified by gating on auto^-^Zombie Aqua-CD3+CD14-CD8+IFNγ+ cells, and proliferating CD8+ T cells were identified by gating on auto^-^SYTOX Blue^-^CD3+CD14-CD8+CFSE^low^ cells.

### 2.12 Statistical analysis

All statistical analyses were performed using Graphpad Prism 9.0.2 (San Diego, CA, USA). Data are shown as the mean ± standard deviation (SD) with at least three replicates, unless otherwise specified. Significances were calculated via Kruskal-Wallis test with Dunn’s post-hoc test. Difference between groups were calculated using two-way analysis of variance (ANOVA) and Tukey’s post-hoc correction. Statistical *p*-values ≤ 0.05 were considered as statistically significant.

## 3 Results

### 3.1 Physiochemical characterization of SPIONs

Citrate-coated SPIONs were synthesized as previously described, and the magnetic susceptibility, hydrodynamic Z-average size, polydispersity index (PDI) and zeta potential of the two SPION batches were investigated (**Table 1**). The two individual batches had similar characteristics and were therefore used in all experiments. Endotoxins were not detected in any batch (data not shown).

**Table 1:** Physicochemical characterization of SPIONs.

Physiochemical Feature	Batch 1	Batch 2
Magnetic susceptibility × 10^-3^ (a.u.)	4.10 ± 0.00	4.19 ± 0.00
Z-average size in H_2_O (nm)	53 ± 0.2	49 ± 0.3
PDI (a.u.)	0.152 ± 0.007	0.168 ± 0.002
Zeta potential at pH 7.3 (mV)	-51.8 ± 0.3	-39.5 ± 2.3

### 3.2 SPION-loading has no impact on T cell mechanics

**Figure 1:**
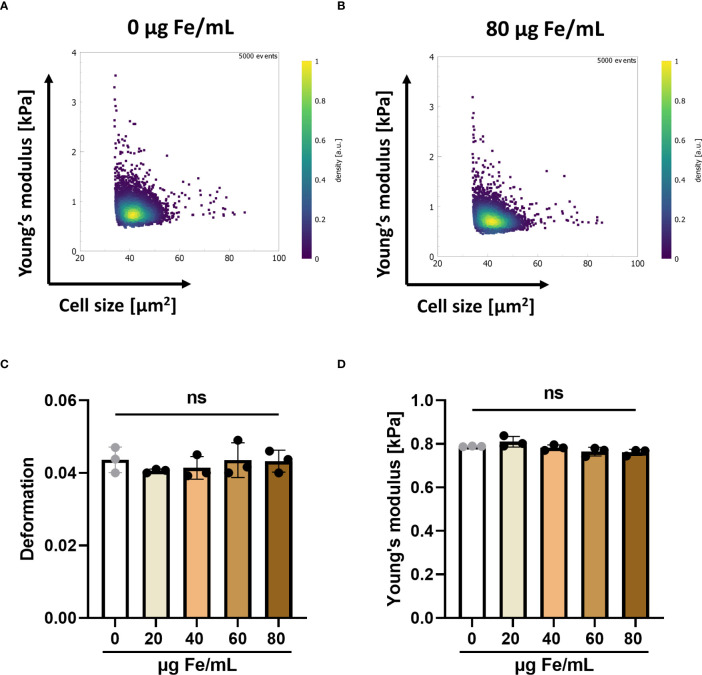
SPION-loading has no effect on the mechanical properties of T cells mechanical properties. Primary human T cells were loaded with up to 80 µg Fe/mL SPIONs overnight, while controls only received dH_2_O. On the following day, cells were analyzed using RT-DC. Typical scatter plots showing cell cross-sectional area and Young’s modulus of 4,000 – 5,000 cells each of **(A)** unloaded T cells and **(B)** SPION-loaded T cells. Median values of **(C)** deformation and **(D)** Young’s modulus. Experiments were performed with samples of 3 donors, shown are the mean values for every donor Significances were calculated using a Kruskal-Wallis test with Dunn’s post-hoc test. ns, non-significant.

T cell deformability is an important factor in the ability of T cells to infiltrate the stiff matrix of solid tumors (41, 42). To investigate the influence of SPIONs on the mechanical phenotype of T cells, we performed RT-DC with T cells, which had been loaded with various SPION concentrations. We analyzed the cell deformation, obtained from images as the cells passed through a microfluidic channel, and the cellular Young’s modulus, a measure of the compressive stiffness of the cells, derived from the cell deformation and cell size. We did not detect a significant impact of SPION-loading on the median deformation and median Young’s modulus of loaded T cells compared to the unloaded controls (**Figure 1**).

### 3.3 Electroporation increases SPION uptake and magnetic controllability in Jurkat cells

For the transient expression of an antigen-specific TCR, we introduced mRNA into T cells. For that, cells were electroporated with a square wave pulse, causing a transient permeabilization of the plasma membrane, which enables the passage of mRNA into the cells. Likewise, an increased nanoparticle uptake has been previously shown after electroporation (43). Despite not being phagocytic cells, we previously showed that SPIONs do not simply attach to the plasma membranes of primary human T cells, but are taken up intracellularly (23). Thus, we investigated, whether the uptake of SPIONs can be increased if SPIONs are applied to cells after electroporation, when the plasma membrane pores are not properly sealed.

**Figure 2:**
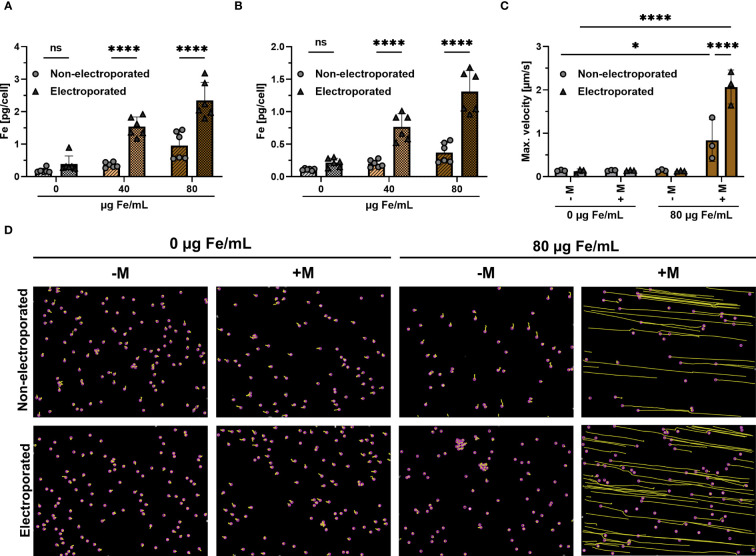
Influence of electroporation on cellular iron uptake and magnetic attraction. Jurkat cells were loaded with either 40 or 80 µg Fe/mL SPIONs, after having received electroporation or not. Controls received only dH_2_O. Cellular iron content was measured after **(A)** 4 h or **(B)** 24 h after SPION-loading using AES. **(C, D)** Jurkat cells, which were loaded with SPIONs, were stained with Hoechst 33342 and then seeded in an ibidi µ-slide to which a magnet was added or not. The Jurkat cells were then imaged over a period of 10 min by microscopy at a distance of 5 mm from the magnet with a magnetic field gradient of 152 mT. **(C)** Maximum velocity of cells towards the magnet was calculated via ImageJ Plugin TrackMate **(D)** Migration paths of SPION-loaded Jurkat cells. The path is depicted in yellow, while the cells are highlighted with a purple circle. Experiments were performed in triplicate samples of 3 **(C)** or 6 replicates **(A, B)**, shown are the mean values for every biological replicate. Significances were calculated using a 2way ANOVA with a post-hoc Tukey correction. ns, non-significant; *p ≤ 0.05; ****p ≤ 0.0001; - M: no addition of a magnet; + M: addition of a magnet; Max.: Maximum.

Jurkat cells were electroporated and subsequently loaded with SPIONs for either 4 h or 24 h. Non-electroporated Jurkat cells with SPIONs served as controls. Subsequently, the amount of cellular iron was measured using atomic emission spectroscopy. Four hours after loading, Jurkat cells without SPIONs showed iron background levels below 0.2 pg Fe/cell, with 40 or 80 µg/mL SPION incubation, cellular iron contents increased to 0.4 or 0.6 pg Fe/cell. When the SPIONs were added after electroporation, a significantly higher iron mount of 1.3 or 2.4 pg Fe/cell was achieved (**Figure 2A**). Incubation of the SPION-loaded cells for a further 20 h approximately halved the iron content per cell under all conditions, reflecting the distribution of the particles to the daughter cells during cell division (**Figure 2B**). After electroporation, the absolute cell number was reduced compared to the untreated controls. However, the cells that survived electroporation were after nanoparticle loading as vital as the untreated control cells (**Supplementary Figure 1**).

Next, we analyzed the dependence of the magnetic attraction of the cells on the intracellular iron content for cells. Jurkat cells were loaded for 4 h with 80 µg Fe/mL SPIONs with or without a prior electroporation step. The cells were then added to a µ-slide, to which a neodymium magnet was added in a 5 mm distance, resulting in a magnetic field gradient of approximately 152 mT. Only SPION-loaded Jurkats under the influence of the magnetic field gradient moved in the slides, whereas electroporated cells moved significantly faster, with a maximum speed of 2.0 µm/s, compared to 0.6 µm/s for non-electroporated cells (**Figures 2C, D**). We conclude that an electroporation prior to SPION-loading increases SPION-uptake, which improves magnetic guidability.

### 3.4 SPION-loading does not impact the functionality of an exogenous or endogenous TCR

#### 3.4.1 SPION-loaded T cells express activation markers and proliferate after stimulation by an exogenous T cell receptor

**Figure 3:**
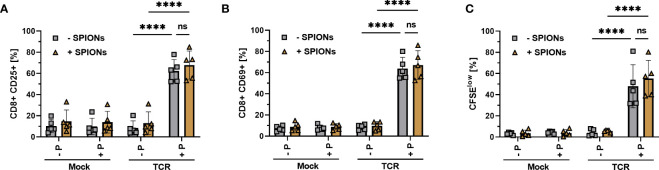
Activation and proliferation of SPION-loaded MelanA specific TCR-T cells. Human T cells were isolated from healthy donors and received no mRNA (Mock) or mRNA coding for an MelanA- specific TCR (TCR). Directly after electroporation, T cells were incubated with 80 µg Fe/mL of SPIONs (+SPIONs) or dH_2_O (-SPIONs) for 4 h. For analysis of proliferation **(C)**, the T cells were stained with 5 µM CFSE after 4 h. T cells were incubated overnight **(A, B)** or for 72 h **(C)** with T2A1 cells, which had previously received either no peptide (-P) or MelanA (+P) for two hours. After incubation, T cells were stained and analyzed by flow cytometry for the expression of **(A)** CD25, **(B)** CD69 and **(C)** fluorescence intensity of CFSE. Experiments were performed in duplicate samples of 5 donors, shown are the mean values for every donor. Significances were calculated using a 2-way ANOVA with a post-hoc Tukey test. ns, non-significant; ****p< 0.0001.

For successful adoptive T cell therapy, it is mandatory that T cells retain their ability to interact with their specific antigens via an exogenous TCR. We previously showed that the loading of primary human T cells with SPIONs did not interfere with their polyclonal activation by human CD3/CD28/CD2 T cell activators (22, 23). To analyze the antigen-specific T cell response, primary human T cells were transfected with a TCR specific for the HLA-A*0201-restricted peptide MelanA via mRNA electroporation (44). The antigen-specific response to the MelanA peptide presented on T2A1 cells was analyzed. Mock-electroporated T cells and T2A1 cells that had not received MelanA, served as controls. The antigen-specific CD8+ T cell response was investigated by detecting the activation markers interleukin-2 receptor subunit alpha (CD25) (**Figure 3A**) and CD69 (**Figure 3B**), with the latter being a marker for early activation (45). T cell proliferation (**Figure 3C**) was investigated by measuring the reduction in cellular CFSE fluorescence intensity after 72 h (46). Gating as well as representative blots for CD69 and CD25 expression and T cell proliferation of unloaded and SPION-loaded TCR-expressing T cells in the presence of MelanA-T2A1 presenting cells are depicted in **Supplementary Figures 2, 3**.

We did neither detect significant expression of CD25 and CD69 nor proliferation in all mock-electroporated conditions or mRNA-electroporated cells without the addition of the MelanA peptide. T cells electroporated with mRNA encoding for the MelanA receptor, however significantly expressed CD25 and CD69 in the presence of MelanA peptide and proliferated, as indicated by the decreased CFSE intensity of the cell. Importantly, there was no significant difference between T cells with or without SPION-loading (**Figure 3**).

#### 3.4.2 SPION-loaded T cells express and secrete cytokines after antigen-specific stimulation via an exogenous T cell receptor

**Figure 4:**
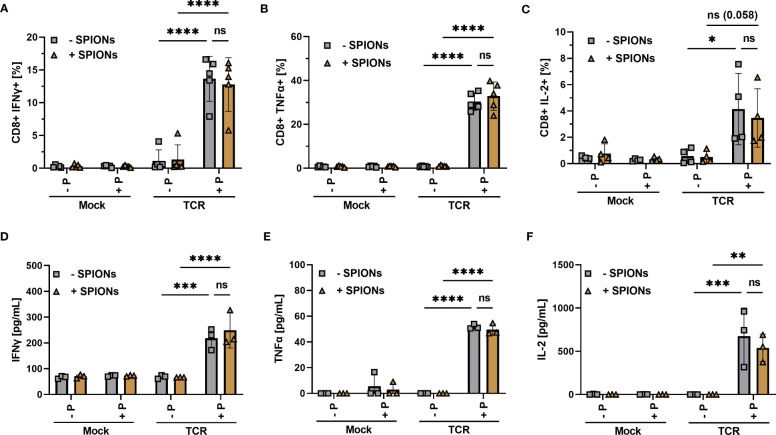
Cytokine production of SPION-loaded MelanA-specific TCR-T cells. Human T cells were isolated from healthy donors and received no mRNA (Mock) or mRNA coding for an MelanA-specific TCR (TCR). After electroporation, T cells were incubated with 80 µg Fe/mL of SPIONs (+SPIONs) or dH_2_O (-SPIONs) for 4 h. T cells were then incubated overnight with T2A1 cells, which had received either no peptide (-P) or MelanA (+P) for two hours prior. Intracellular cytokine expression was measured in flow cytometry; **(A)** IFNγ, **(B)** TNFα, and **(C)** IL-2. **(D–F)** Supernatants were investigated via ELISA; **(D)** IFNγ, **(E)** TNFα, and **(F)** IL-2. Experiments were performed in duplicate samples of 3 **(D–F)** or 5 donors **(A-C)**, shown are the mean values for every donor. Significances were calculated using a 2-way ANOVA with a post-hoc Tukey test. ns, non-significant; *p ≤ 0.05; **p ≤ 0.01; ***p ≤ 0.001; ****p< 0.0001.

Inflammatory cytokine production and secretion are indispensable for a successful anti-tumor response (47). T cells were loaded with SPIONs, stimulated as described before and then analyzed for intracellular (**Figures 4A–C**) and secreted cytokines (**Figures 4D–F**). Gating as well as representative blots for cytokine production of unloaded and SPION-loaded TCR-expressing T cells in the presence of MelanA-T2A1 presenting cells are depicted in **Supplementary Figures 4, 5**.

We observed a significant increase in intracellular IFNγ and TNFα in MelanA-specific TCR-expressing T cells after co-incubation with MelanA-presenting T2A1 cells, compared to the controls (**Figures 4A, B**). For IL-2 the difference was visible, but only missed formal significance (p = 0.058) (**Figure 4C**). When we investigated the supernatants for released cytokines via ELISA, we found a significant increase in IFNγ, TNFα, and IL-2 for MelanA-specific TCR-expressing T cells, co-incubated with T2A1 cells presenting MelanA, compared to the other conditions (**Figures 4D–F**). Most importantly, in line with our activation and proliferation data, we did not observe a decrease in cytokine expression or release in SPION-loaded cells (**Figure 4**).

**Figure 5:**
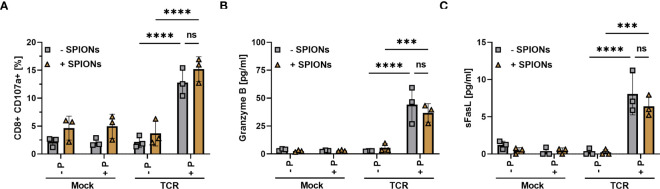
Degranulation and release of Granzyme B and sFasL by SPION-loaded MelanA-specific TCR-T cells. Human T cells were isolated from healthy donors and received no mRNA (Mock) or mRNA coding for an MelanA-specific TCR (TCR). After electroporation, T cells were incubated with 80 µg Fe/mL of SPIONs (+SPIONs) or dH_2_O (-SPIONs) for 4 h. The T cells were then incubated overnight with T2A1 cells, which had received either no peptide (-P) or MelanA (+P) for two hours. **(A)** CD107a in secretory vesicles was analyzed by anti-human CD107a antibody staining in flow cytometry; **(B, C)** pro-apoptotic proteins were investigated in the supernatants via a bead-based multiplex assay in flow cytometry. Experiments were performed in duplicate samples of 3 donors, shown are the mean values for every donor. Significances were calculated using a two-way ANOVA with a post-hoc Tukey test. ns, non-significant; ***p ≤ 0.001; ****p< 0.0001.

TCR-induced killing of target cells is mediated mainly by the secretion of lytic granules, which involves the fusion of the granule membrane with the cytoplasmic membrane of the T cell, resulting in surface exposure of lysosomal-associated proteins, which are typically present in the membrane of lysosomal vesicles, such as CD107a (48, 49). When we used the transmembrane protein CD107a as a marker for degranulation and cytotoxic activity, we detected a significant increase in the frequency of CD107-stained CD8+ T cells after stimulation with MelanA-presenting T2A1 cells (**Figure 5A**), compared to the other control conditions, indicating degranulation. Gating as well as representative blots for CD107a-expression of unloaded and SPION-loaded TCR-expressing T cells in the presence of MelanA-T2A1 presenting cells are depicted in **Supplementary Figure 6**.

Furthermore, we investigated the supernatant via a bead-based immunoassay for pro-apoptotic proteins such as Granzyme B (**Figure 5B**) and soluble Fas ligand (sFasL) (**Figure 5C**) (50–52). In the supernatant of mock-electroporated T cells or TCR- electroporated cells without MelanA-presenting T2A1 cells, we did not detect the release of Granzyme B and sFasL. However, there was a significant increase in Granzyme B and sFasL levels in the supernatant of TCR-electroporated T cells in the presence of MelanA (**Figures 5B, C**). Again, loading of the T cells with SPIONs n did not significantly affect CD107a-exposure nor Granzyme B- and sFasL-release.

In summary, SPION-loading did not significantly affect the degranulation of T cells or the release of soluble mediators such as cytokines (**Figures 4D–F**) or apoptosis-inducing factors (**Figures 5B, C**) after antigen-specific stimulation via an exogenous TCR.

#### 3.4.3 SPION-loaded T cells retain the ability to perform antigen-specific tumor lysis

**Figure 6:**
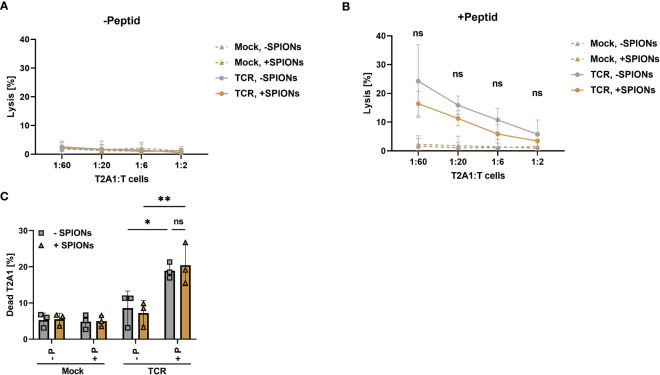
Antigen specific cytotoxicity-mediated by SPION-loaded MelanA-specific TCR-T cells. Human T cells were isolated from healthy donors and received no mRNA (Mock) or mRNA coding for an MelanA-specific TCR (TCR). After electroporation, T cells were incubated with 80 µg Fe/mL of SPIONs (+SPIONs) or dH_2_O (-SPIONs) for 4 h **(A, B)** cytotoxicity was determined by chromium release assay with T cells which had been co-incubated with T2A1 cells for 4 h, which had received either **(A)** no peptide (-P) or **(B)** MelanA (+P) for one hour. **(C)** The amount of dead T2A1 cells after co-incubation with MelanA-specific TCR-expressing T cells was determined after 16 h of co-incubation in flow cytometry after staining with a viability dye. Experiments were performed in technical triplicates of 4 donors **(A, B)** or duplicates of 3 donors **(C)**, **(A, B)** shows the mean values of all donors, **(C)** each individual donor as symbols and the overall mean as bars. Significances were calculated using a two-way ANOVA with a post-hoc Tukey test. ns, non-significant; *p< 0.05; **p ≤ 0.01.

Cytolytic capabilities of SPION-loaded MelanA-specific T cells was assessed after co-incubation with peptide-loaded T2A1 cells for 4 h via chromium-release assay and after 16 hours via flow cytometry. Release of intracellular chromium was only observed after co-incubation of TCR-expressing T cells with MelanA presenting T2A1 cells. Additionally, with increasing ratio of T cells to T2A1 cells, more cells were lysed. The loading of TCR-expressing T cells with SPIONs slightly decreased the level of chromium release, but not significantly (**Figures 6A, B**).

Antigen-specific cytotoxity was also analyzed after 16 h of co-incubation of T cells with T2A1 cells by flow cytometry. Gating as well as representative histograms for the viability of T2A1 cells after co-incubation with unloaded and SPION-loaded TCR-expressing T cells are depicted in **Supplementary Figure 7**. In line with the data obtained from the chromium release assay, only TCR expressing T cells killed MelanA-presenting T2A1 cells (**Figure 6C**). In the absence of MelanA peptide or with mock-electroporated T cells, no increase in cell death occurred.

#### 3.4.4 SPION-loaded T cells retain their ability to recognize antigen via their natural T cell receptor

After analyzing the antigen-specific TCR response with an exogenous TCR, expressed after mRNA electroporation (**Figures 3**–**6**), we investigated the primary immune response of T cells with the natural TCR. We isolated T cells from CMV-seropositive donors, which represent >50% of the German population (53). Co-cultured monocytes and T cells from the same donor were stimulated with either the CMV-derived peptide pp65, or MOG peptide, or received no peptide as a stimulation control.

Gating as well as representative blots for intracellular IFNγ expression or proliferation of unloaded and SPION-loaded CMV-specific CD8+ T cells are depicted in **Supplementary Figures 8, 9.**

**Figure 7:**
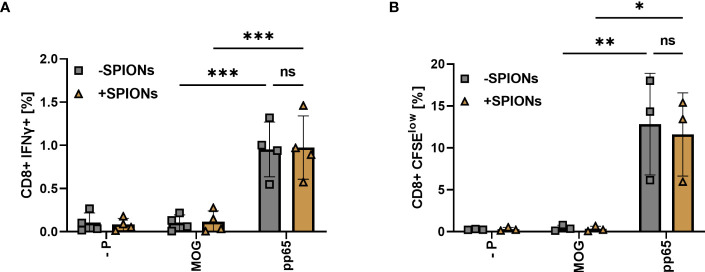
T cell response of SPION-loaded T cells to CMV peptide pp65 mediated by an endogenous TCR. T cells isolated from CMV-seropositive individuals were incubated with SPIONs (+SPIONs) overnight or dH2O (-SPIONs). The T cells were then stimulated with either the CMV-derived peptide pp65, MOG or no peptide (- P) via monocytes. **(A)** After 11 h, intracellular IFNγ content and **(B)** after 5 d CFSE fluorescence intensity of CD8+ T cells was investigated by flow cytometry. Experiments were performed in duplicate samples of 3 **(B)** or 4 donors **(A)**, shown are the mean values for every donor. Significances were calculated using a two-way ANOVA with a post-hoc Tukey test. Ns, non-significant; *p ≤ 0.05; **p ≤ 0.01; ***p ≤ 0.001.

Co-incubation of T cells with monocytes and pp65 resulted in an increase in CD8+ T cells intracellularly expressing IFNγ, compared to T cells stimulated with MOG or no peptide. No significant difference was observed between SPION-loaded T cells and T cells without SPIONs (**Figure 7A**). T cell proliferation after stimulation was investigated using CFSE staining. With the pp65 peptide, increased numbers of proliferating T cells were observed, regardless of whether they were loaded with SPIONs (**Figure 7B**).

## 4 Discussion

The infiltration of T cells has been shown to be predictive for the prognosis and treatment options for patients with several types of cancer (54). Patients with T cell-infiltrated tumors, referred to as “hot”, tended to have better clinical outcomes (55, 56). Enhancing T cell infiltration into “cold” tumors, excluded and/or ignored from immune cells, may increase response rates to immunotherapy and improve survival. The adoptive transfer of tumor-specific T cells in an attempt to increase the number of tumor-infiltrating T cells is still challenging due to several obstacles, such as the immune suppressive microenvironment of the tumor, hampering T cell trafficking and infiltration into the tumor.

To increase the number of T cells in the tumor region, we developed SPIONs with a citrate coating to equip T cells with magnetic guidability (20, 21). A variety of physicochemical factors are known to influence the nanoparticle uptake such as size, shape, surface charge, surface hydrophobicity/hydrophilicity and surface functionalization (57). When SPION-loaded cells are applied intravascularly and magnetically pulled to the desired region, we assume that the magnetic attraction first causes an increase in the attachment to the vessel wall, which still has to be passed by the T cells. The magnetic force might support this process; however, cells must deform to adhere to the endothelium (“rolling”) and to squeeze through the dense packaging formed by the endothelial cells (“diapedesis”). It has been previously reported that nanoparticle interactions with cells can impair cell mechanics such as cell adhesion and cell stiffness (58). Cell stiffness refers to the resistance of a cell to an externally induced deformation, which is commonly expressed by the Young’s modulus, the ratio between the applied mechanical stress and the resulting applied strain (59). Iron oxide nanoparticles applied as contrast agents, such as Resovist^®^ or Endorem^®^, can have an effect on the actin cytoskeleton and microtubule network, when the intracellular iron concentrations (> 40 pg Fe/cell) are very high, which has already been shown in C17.2 neural progenitor cells and human blood outgrowth endothelial cells (60). In leukocytes, gadolinium particles applied as contrast agents also caused cell stiffening, as determined by deformability cytometry (61). Using deformability cytometry, we showed that SPION loading does not alter the mechanical properties of T cells, as quantified by the Young’s modulus (**Figure 1**), which might be due to the relatively low cellular nanoparticle concentrations around 1 pg Fe/cell (23).

Because T cells are not phagocytic, their loading efficacy is typically low. However, magnetic guidability depends on the amount of cellular SPIONs. Methods such as functionalization of particles with virus-derived peptides have been used to increase the loading of T cells with iron oxide nanoparticles (62, 63). Also, electroporation has been employed to enhance the uptake of various nanoparticles into cells (64). In line with these reports, we investigated whether electroporation prior to SPION incubation can increase nanoparticle uptake, because we used this procedure to transfect cells with mRNA for transient expression, as used for adoptive T cell therapy. We found that with a prior electroporation step, nanoparticle uptake can be more than doubled, which might be due to penetration through the temporary pores formed during the electroporation process (**Figures 2A, B**). 24 h of incubation after nanoparticle loading, the iron amount decreased in the Jurkat cell line, which was mainly due to cell division and distribution of the nanoparticles to the daughter cells, coinciding with the findings of other studies (65). In non-dividing primary cells, we found that particles were lost during prolonged incubation for 72 h, potentially caused by mechanisms other than cell division (23). We have already shown that SPION-loading allows for the magnetic attraction of cells, even under flow conditions (23, 66). Based on these results, we showed that increased iron loading, achieved by electroporation, improves magnetic attraction, probably being beneficial for magnetic cell targeting in adoptive T cell therapy (**Figures 2C, D**).

SPION-loading has previously been shown not to interfere with antigen-nonspecific stimulation by CD3/CD28/CD2 beads, crosslinking the CD3 and CD28 cell surface ligands, thereby providing the signals required for T cell activation (22). For adoptive cell therapy, tumor-specific T cells are either isolated from tumor-infiltrating lymphocytes or genetically engineered to express tumor-specific TCR (38, 67). The targeted enrichment in the tumor region might improve tumor targeting and reduce on-target/off-tumor toxicity (68). When T cells are loaded with SPIONs for magnetic targeting, it is mandatory for T cells to retain their ability to specifically recognize target cells expressing the appropriate antigen.

Here, we investigated T cells that were transiently electroporated with mRNA encoding for an exogenous TCR, specific for a MelanA peptide (**Figures 3**–**6**). Additionally, we analyzed the antigen-specific response of SPION-loaded T cells isolated from CMV-positive donors, equipped with CMV-specific endogenous TCRs permanently expressed on T cells (7). In both cases, SPION-loading did not impair antigen-specific T cell responses. This was determined by intracellular cytokine production and secretion, activation marker expression, cell proliferation for MelanA-specific electroporated T cells (**Figures 3**-**5**) or by cell proliferation and intracellular IFNγ production for CMV-specific T cells (**Figure 7**). Importantly, SPION loading did not limit cytolytic function of the TCR-T cells (**Figure 6**). In chromium release assay, determined after 4 h of co-incubation of T cells with target cells, we observed a weak reduction of cytotoxicity towards MelanA-presenting T2A1 cells, when TCR-expressing T cells were loaded with SPIONs, however, this effect was not significant (**Figure 6A**). When co-incubating the cells for a longer time period (16 hours), the difference between unloaded and SPION-loaded TCR-expressing T cells was abolished. We speculate that the weak effects detected by chromium release assay were either very early effects or only measurable by this very sensitive cytotoxicity detection method (**Figure 6**).

In a murine system, others investigated the conservation of antigen-specific CD8+ T cell responses of ovalbumin-specific T cells of OT 1 mice with EG7-OVA cells, expressing the OVA peptide presented by H-2Kb on its surface after magnetic nanoparticle loading (26). In line with our findings, they found no influence of SPION-loading on conjugation kinetics, IFNγ production, and cytolytic activity. Interestingly, nanoparticles did not alter the basal degranulation of the cells, but after co-incubation with the EG7-OVA target cells, degranulation was dose-dependently increased in the presence of the nanoparticles (26). For NK cells, also an increase in spontaneous and stimulated degranulation has been described after loading with iron oxide nanoparticles (25). We observed a weak, but not significant increase in CD107a expression in CD8+ T cells in the presence of SPIONs in all conditions (**Figure 5A**). These and our findings suggest that an increase in degranulation by iron oxide nanoparticles could be a general phenomenon of cytotoxic cells (69).

In line with our findings on deformability, Sanz-Ortega et al. did not detect alterations in adhesion, transmigration, and chemotaxis by magnetic nanoparticles (26). Magnetic nanoparticles attached to cells or taken up into the cytoplasm in the presence of a magnetic field can also provide mechanical stimulation to cells, inducing F-actin polymerization and cell asymmetry related to nanoparticle-induced tension (70). These effects of SPIONs on T cells in the presence of a magnetic field still need to be elucidated. Since the magnetic force in our case was applied only for a short time of 30 min, the effects might only be temporary. Additionally, it remains unclear whether extravasation of SPION-loaded T cells from tumor-supporting vessels into the tumor region is improved under the influence of an external magnetic field. Sanz-Ortega et al. observed the retention of murine T cells in tumor-draining lymph nodes in mice; however, it was suspected that cellular aggregation was mediated by the positively charged 3-aminopropyl-triethoxysilane-coated nanoparticles, which we did not observe with our negatively-charged citrate-coated SPIONs (26).

Infiltration and persistence in solid tumors are still among the most challenging aspects of adoptive T cell therapy. We hypothesized that these problems can be addressed by the functionalization of T cells with SPIONs for magnetic accumulation in the tumor region. We found that SPION-loading did not affect cellular stiffness, which is an important factor for diapedesis and movement through the extracellular matrix. We were also able to increase the cellular iron content by electroporation, which directly improved magnetic guidability. Additionally, we observed no significant impact of SPION-loading on T cell activation, proliferation, cytokine production, and tumor cell lysis mediated by an exogenous TCR or its original endogenous TCR. These findings provide new insights for the functionalization of T cells with SPIONs for the improvement of adoptive T cell therapies for solid tumors.

## Data availability statement

The original contributions presented in the study are included in the article/**Supplementary Material**. Further inquiries can be directed to the corresponding author.

## Ethics statement

The studies involving humans were approved by Ethics Committee of the Friedrich-Alexander-Universität Erlangen-Nürnberg reference numbers 257_14 B, 346_18B and 60-21B. The studies were conducted in accordance with the local legislation and institutional requirements. The participants provided their written informed consent to participate in this study.

## Author contributions

FP, JD, NS, VB, and CJ designed the experiments. FP, HU, JD, NS, VB, LC, PS, RS and CJ generated the data, developed the protocols, and performed the experiments. FP performed the statistical analyses. FP and CJ prepared the manuscript. FP and CJ contributed to the discussion of the data. CJ and CA supervised the study. All authors reviewed, edited, and commented on the manuscript. All authors contributed to the article and approved the submitted version.

## Supplementary material

Supplementary Figure 1 The effect of electroporation and SPION loading on cellular viability. Jurkat T cells were either electroporated with two square-wave pulses of 500 V/cm for 2 ms at 0.1 s intervals (+E) or received none (-E). Immediately afterwards, Jurkat cells were loaded with either 40 or 80 μg Fe/mL SPIONs or only dH2O as a control (0) and analyzed for viability after **(A)** 4 h or **(B)** 24 h by flow cytometry. Significances were calculated using a 2way ANOVA with Geisser-Greenhouse correction and a Tukey’s post hoc test. Experiments were performed in duplicates of 7 replicates. Ns, non-significant; AxV- PI-, viable; AxV+ PI-, apoptotic; AxV+ PI+, necrotic.

Supplementary Figure 2 Analysis of CD25 or CD69 expressing CD8+ T cells. **(A)** Gating strategy. Lymphocytes were gated on size and granularity. Apoptotic cells were excluded via Annexin V and cytotoxic T cells identified via CD8 staining. Activated CD8+ T cells were identified via CD25 or CD69 expression. **(B)** Representative blots for the following conditions: TCR, TCR+SPIONs; TCR+P, TCR+P+SPIONs; FSC, Forward Scatter; SSC, Side Scatter; AxV, Annexin V.

Supplementary Figure 3 Analysis of proliferating CD8+ T cells. **(A)** Gating strategy. After 72h, lymphocytes were gated on size and granularity. Apoptotic cells were excluded via Annexin V and cytotoxic T cells identified via CD8 staining. Proliferating CD8+ T cells were identified via low CFSE fluorescence intensity. **(B)** Representative blots for the following conditions: TCR, TCR+SPIONs; TCR+P, TCR+P+SPIONs; FSC, Forward Scatter; SSC, Side Scatter; AxV, Annexin V.

Supplementary Figure 4 Analysis of intracellular IFNγ or TNFα production of CD8+ T cells. **(A)** Gating strategy. Lymphocytes were gated on size and granularity. Viable cytotoxic T cells were gated via fixable viability dye and CD8 staining. Intracellular cytokine production was identified via staining for IFNγ and TNFα. **(B)** Representative blots for the following conditions: TCR, TCR+SPIONs; TCR+P, TCR+P+SPIONs; FSC, Forward Scatter; SSC, Side Scatter; FixV, fixable viability dye.

Supplementary Figure 5 Analysis of intracellular IL-2 production of CD8+ T cells. **(A)** Gating strategy. Lymphocytes were gated on size and granularity. Viable cytotoxic T cells were gated via fixable viability dye and CD8 staining. Intracellular cytokine production was identified via staining for IL-2. **(B)** Representative blots for the following conditions: TCR, TCR+SPIONs; TCR+P, TCR+P+SPIONs; FSC, Forward Scatter; SSC, Side Scatter; FixV, fixable viability dye.

Supplementary Figure 6 Analysis of the degranulation of CD8+ T cells. **(A)** Gating strategy. Lymphocytes were gated on size and granularity. Viable cytotoxic T cells were gated via fixable viability dye and CD8 staining. Degranulating CD8+ T cells were identified via co-culturing of T cells with anti-CD107a antibodies. **(B)** Representative blots for the following conditions: TCR, TCR+SPIONs; TCR+P, TCR+P+SPIONs; FSC, Forward Scatter; SSC, Side Scatter; FixV, fixable viability dye.

Supplementary Figure 7 Analysis of T cell-mediated cytotoxicity. **(A)** Gating strategy. T2A1 cells were identified via forward and sideward scatter and confirmed by CFSE staining. Viable T2A1 cells were identified by gating on CFSE+ Sytox low cells. **(B)** Representative blots for the following conditions: TCR, TCR+SPIONs; TCR+P, TCR+P+SPIONs; FSC, Forward Scatter; SSC, Side Scatter; Viab, Viable.

Supplementary Figure 8 Analysis of intracellular IFNγ of CMV-specific CD8+ T cells. **(A)** Gating strategy. Doublets and cell debris were excluded via forward and sideward scatter, as well as dead cells via SYTOX Blue staining. Auto-fluorescent cells were excluded via a dump channel. IFNγ expressing CD8+ T cells were identified by gating on CD3+CD14-CD8+ cells. **(B)** Representative blots for mononcytes incubated with unloaded or SPION-loaded T cells in the presence of MOG peptide or pp65 peptide. Sytox, SYTOX Blue; Auto, Auto-fluorescent.

Supplementary Figure 9 Proliferation analysis of CMV-specific CD8+ T cells. **(A)** Gating strategy. Doublets and cell debris were excluded via forward and sideward scatter, as well as dead cells via Zombie Aqua staining. Auto-fluorescent cells were excluded via a dump channel. Proliferating CD8+ T cells were identified by gating on CD3+CD14-CD8+ CFSE low cells. **(B)** Representative blots for mononcytes incubated with unloaded or SPION-loaded T cells in the presence of MOG peptide or pp65 peptide. Zombie, Zombie Aqua; Auto, Auto-fluorescent.
