# Supplementary figures and images for: Human T cells loaded with superparamagnetic iron oxide nanoparticles retain antigen-specific TCR functionality
Source: Front Immunol. 2023 Aug 17;14:1223695. doi: 10.3389/fimmu.2023.1223695 (PMC10470061; doi:10.3389/fimmu.2023.1223695)

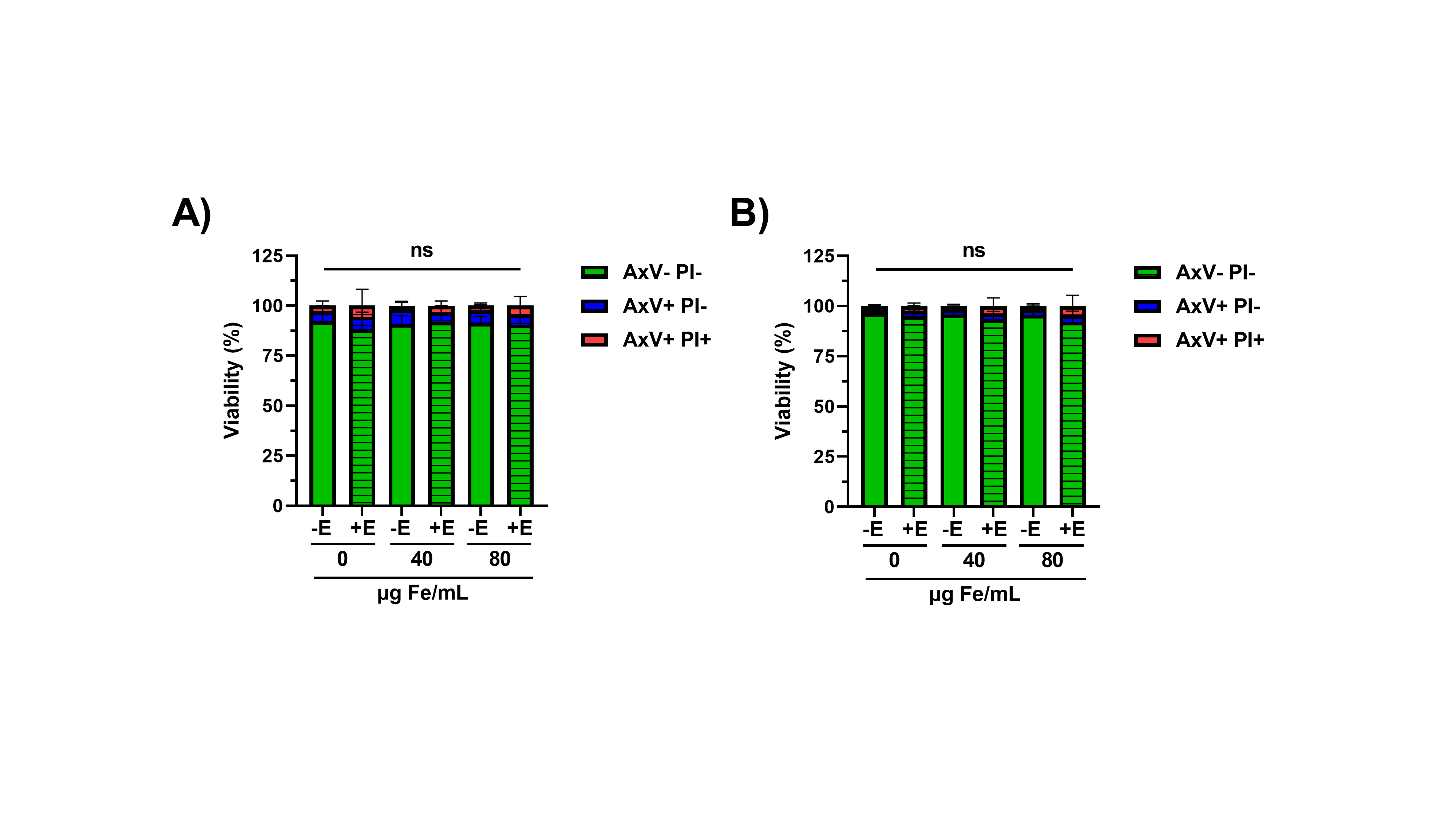

Supplement: Supplementary Figure 1 — The effect of electroporation and SPION loading on cellular viability. Jurkat T cells were either electroporated with two square-wave pulses of 500 V/cm for 2 ms at 0.1 s intervals (+E) or received none (-E). Immediately afterwards, Jurkat cells were loaded with either 40 or 80 μg Fe/mL SPIONs or only dH2O as a control (0) and analyzed for viability after (A) 4 h or (B) 24 h by flow cytometry. Significances were calculated using a 2way ANOVA with Geisser-Greenhouse correction and a Tukey’s post hoc test. Experiments were performed in duplicates of 7 replicates. Ns, non-significant; AxV- PI-, viable; AxV+ PI-, apoptotic; AxV+ PI+, necrotic. [file Image_1.tif]

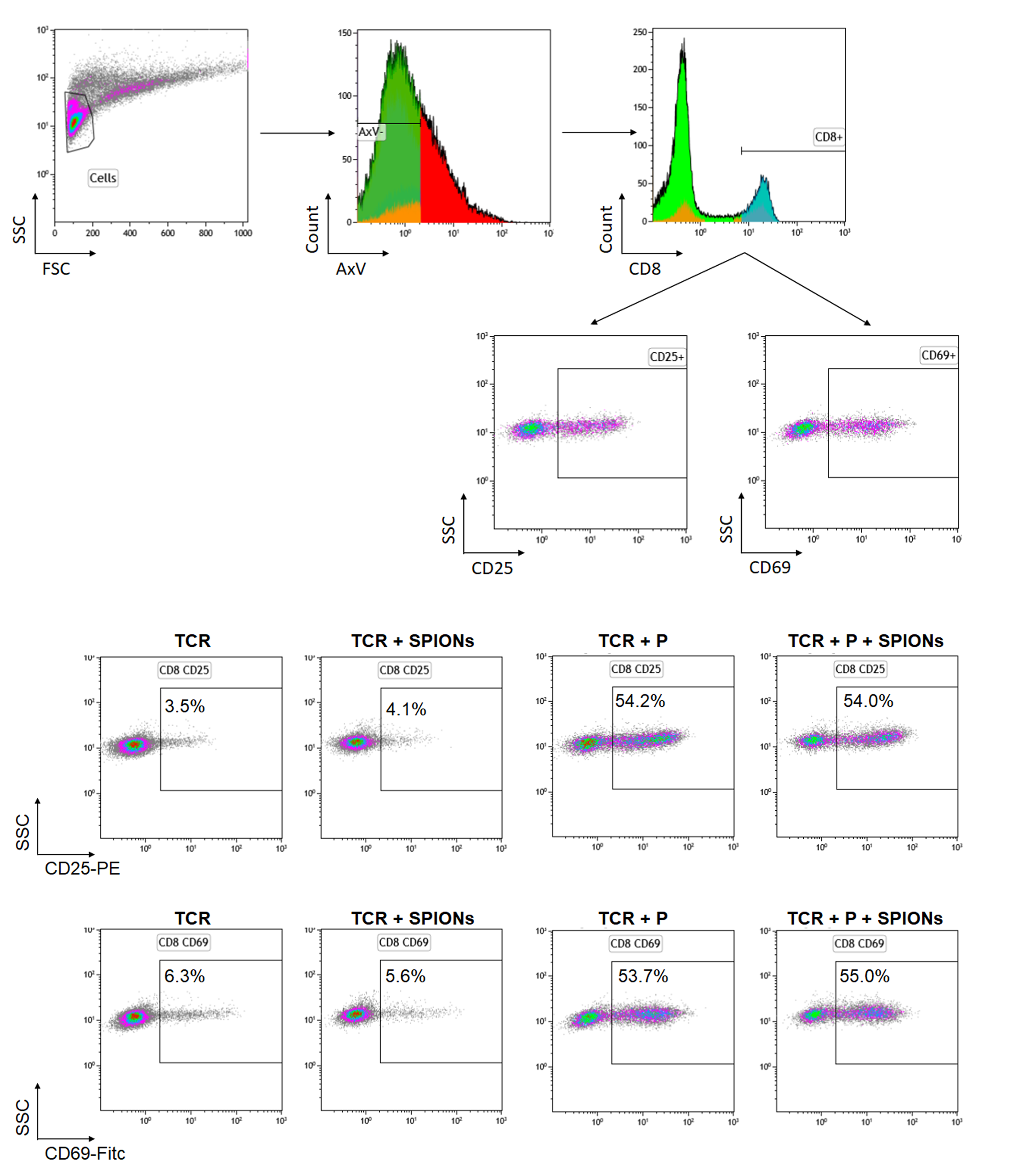

Supplement: Supplementary Figure 2 — Analysis of CD25 or CD69 expressing CD8+ T cells. (A) Gating strategy. Lymphocytes were gated on size and granularity. Apoptotic cells were excluded via Annexin V and cytotoxic T cells identified via CD8 staining. Activated CD8+ T cells were identified via CD25 or CD69 expression. (B) Representative blots for the following conditions: TCR, TCR+SPIONs; TCR+P, TCR+P+SPIONs; FSC, Forward Scatter; SSC, Side Scatter; AxV, Annexin V. [file Image_2.tif]

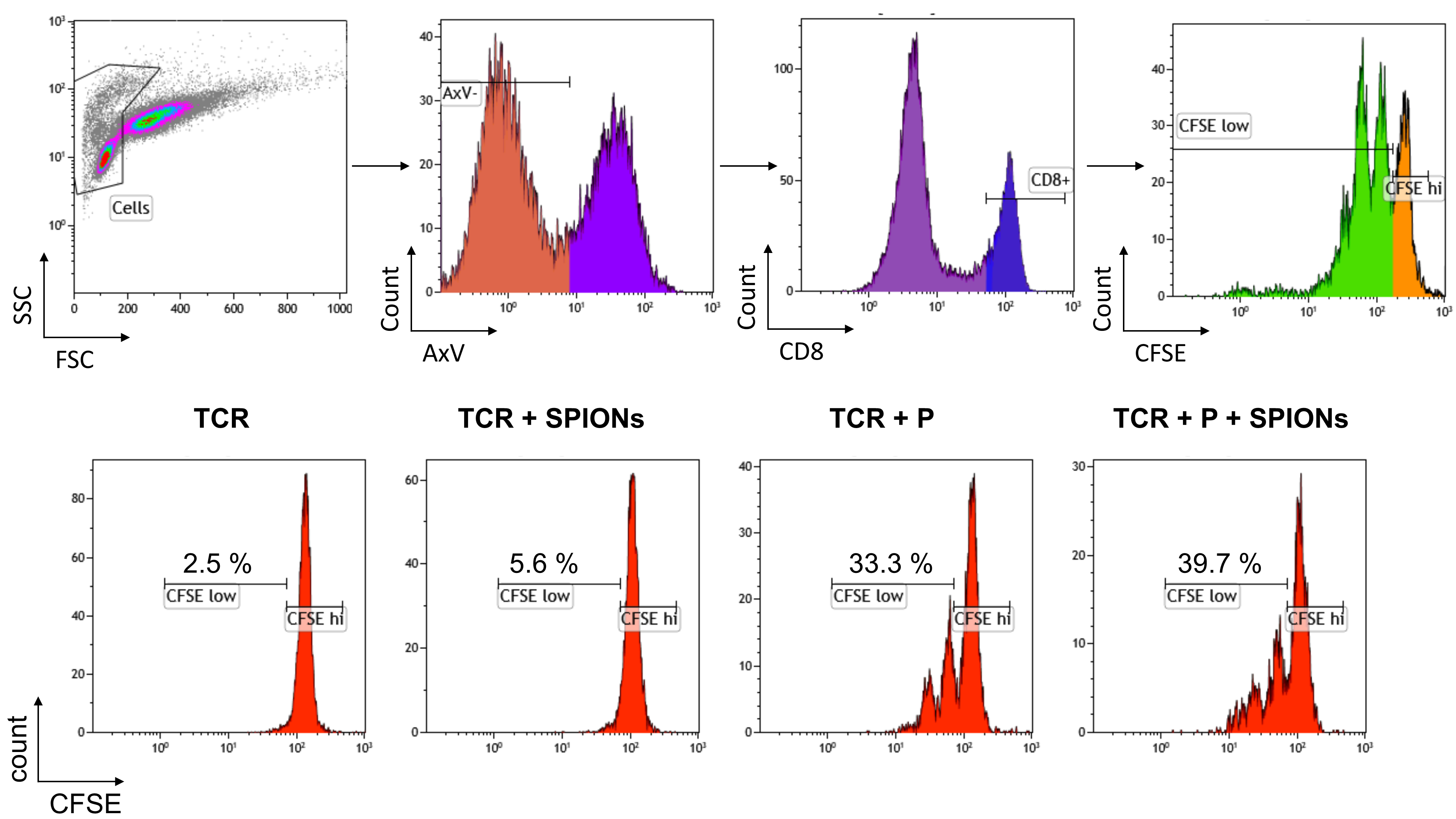

Supplement: Supplementary Figure 3 — Analysis of proliferating CD8+ T cells. (A) Gating strategy. After 72h, lymphocytes were gated on size and granularity. Apoptotic cells were excluded via Annexin V and cytotoxic T cells identified via CD8 staining. Proliferating CD8+ T cells were identified via low CFSE fluorescence intensity. (B) Representative blots for the following conditions: TCR, TCR+SPIONs; TCR+P, TCR+P+SPIONs; FSC, Forward Scatter; SSC, Side Scatter; AxV, Annexin V. [file Image_3.tif]

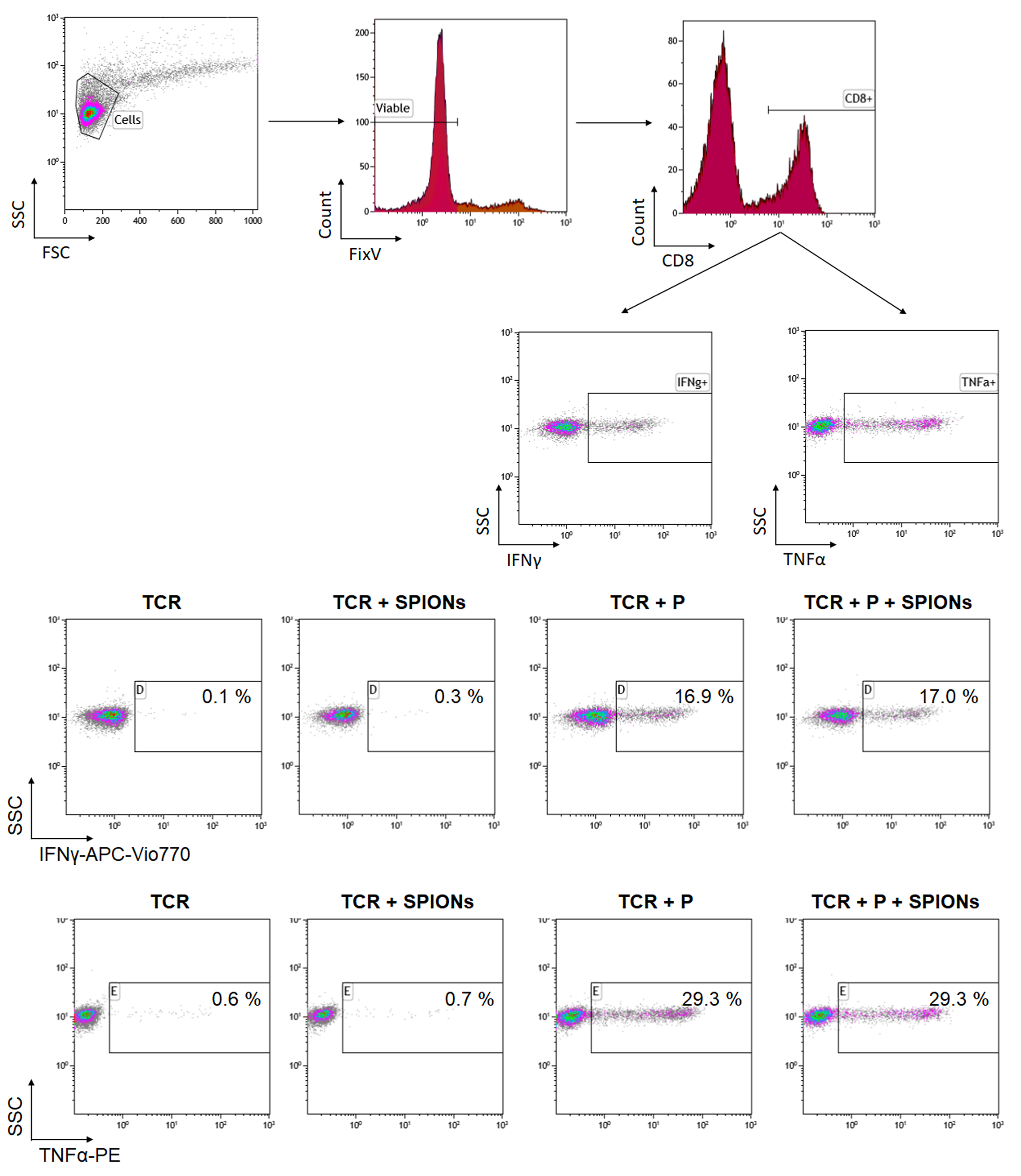

Supplement: Supplementary Figure 4 — Analysis of intracellular IFNγ or TNFα production of CD8+ T cells. (A) Gating strategy. Lymphocytes were gated on size and granularity. Viable cytotoxic T cells were gated via fixable viability dye and CD8 staining. Intracellular cytokine production was identified via staining for IFNγ and TNFα. (B) Representative blots for the following conditions: TCR, TCR+SPIONs; TCR+P, TCR+P+SPIONs; FSC, Forward Scatter; SSC, Side Scatter; FixV, fixable viability dye. [file Image_4.tif]

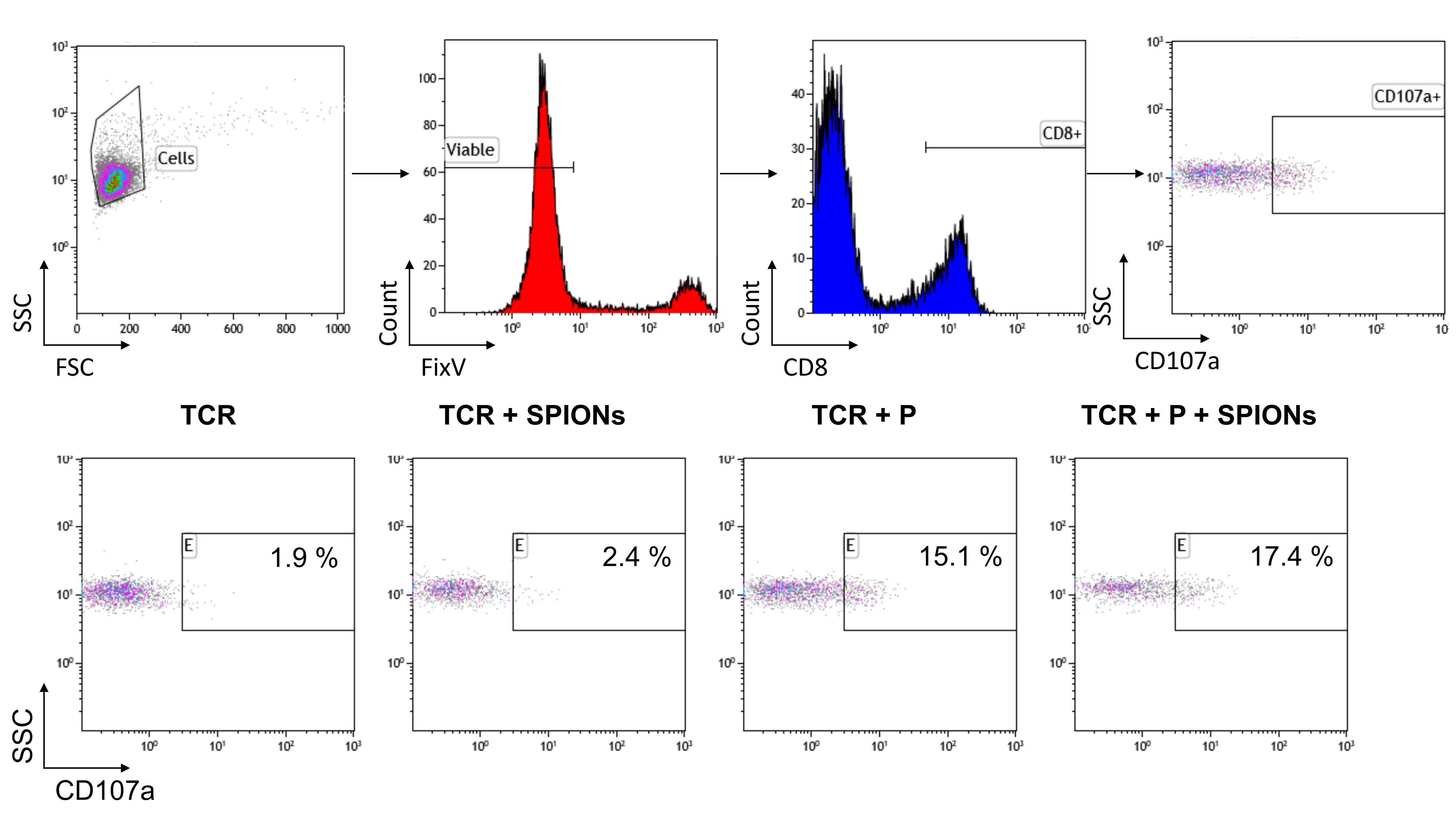

Supplement: Supplementary Figure 6 — Analysis of the degranulation of CD8+ T cells. (A) Gating strategy. Lymphocytes were gated on size and granularity. Viable cytotoxic T cells were gated via fixable viability dye and CD8 staining. Degranulating CD8+ T cells were identified via co-culturing of T cells with anti-CD107a antibodies. (B) Representative blots for the following conditions: TCR, TCR+SPIONs; TCR+P, TCR+P+SPIONs; FSC, Forward Scatter; SSC, Side Scatter; FixV, fixable viability dye. [file Image_6.tif]

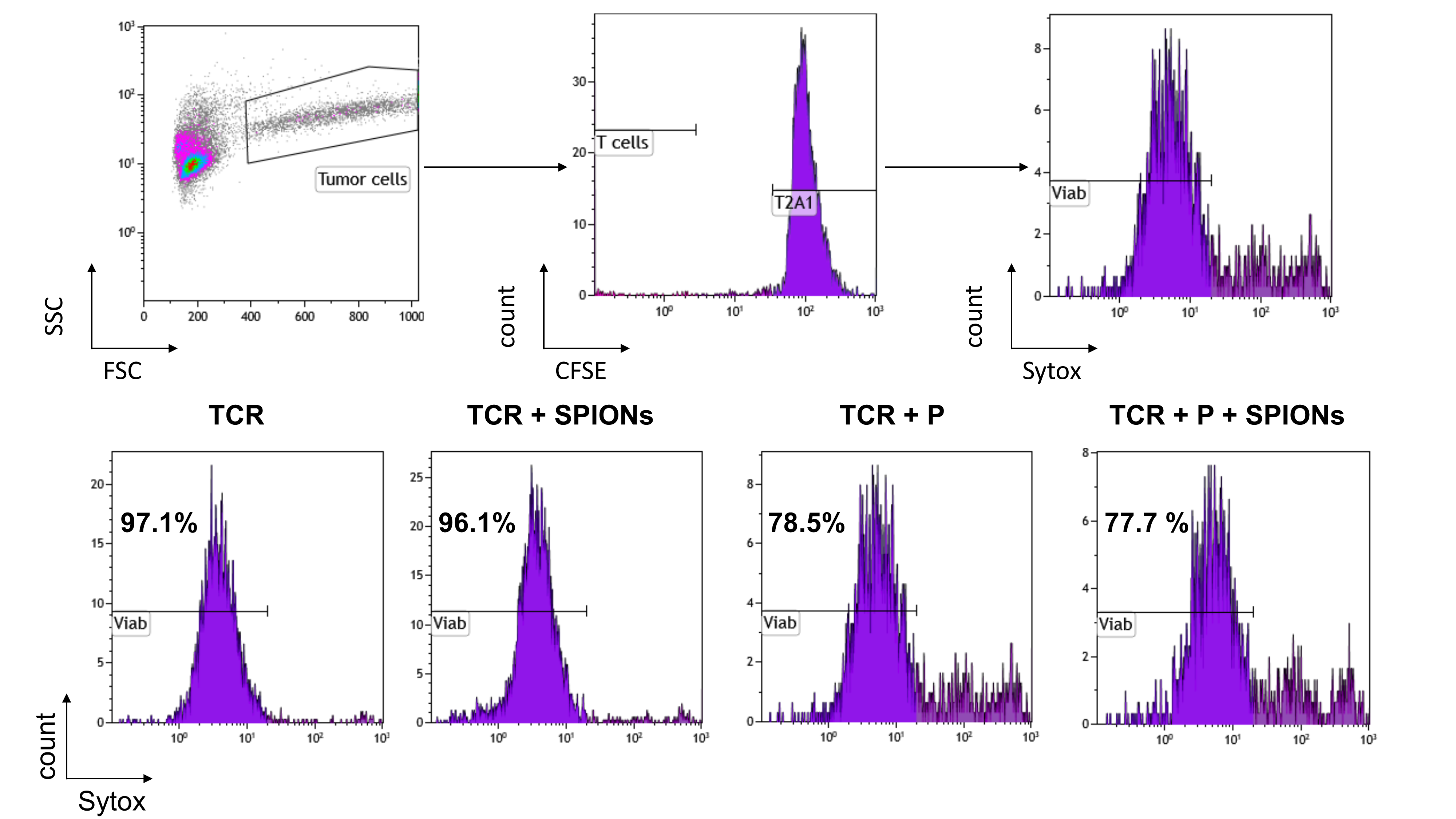

Supplement: Supplementary Figure 7 — Analysis of T cell-mediated cytotoxicity. (A) Gating strategy. T2A1 cells were identified via forward and sideward scatter and confirmed by CFSE staining. Viable T2A1 cells were identified by gating on CFSE+ Sytox low cells. (B) Representative blots for the following conditions: TCR, TCR+SPIONs; TCR+P, TCR+P+SPIONs; FSC, Forward Scatter; SSC, Side Scatter; Viab, Viable. [file Image_7.tif]

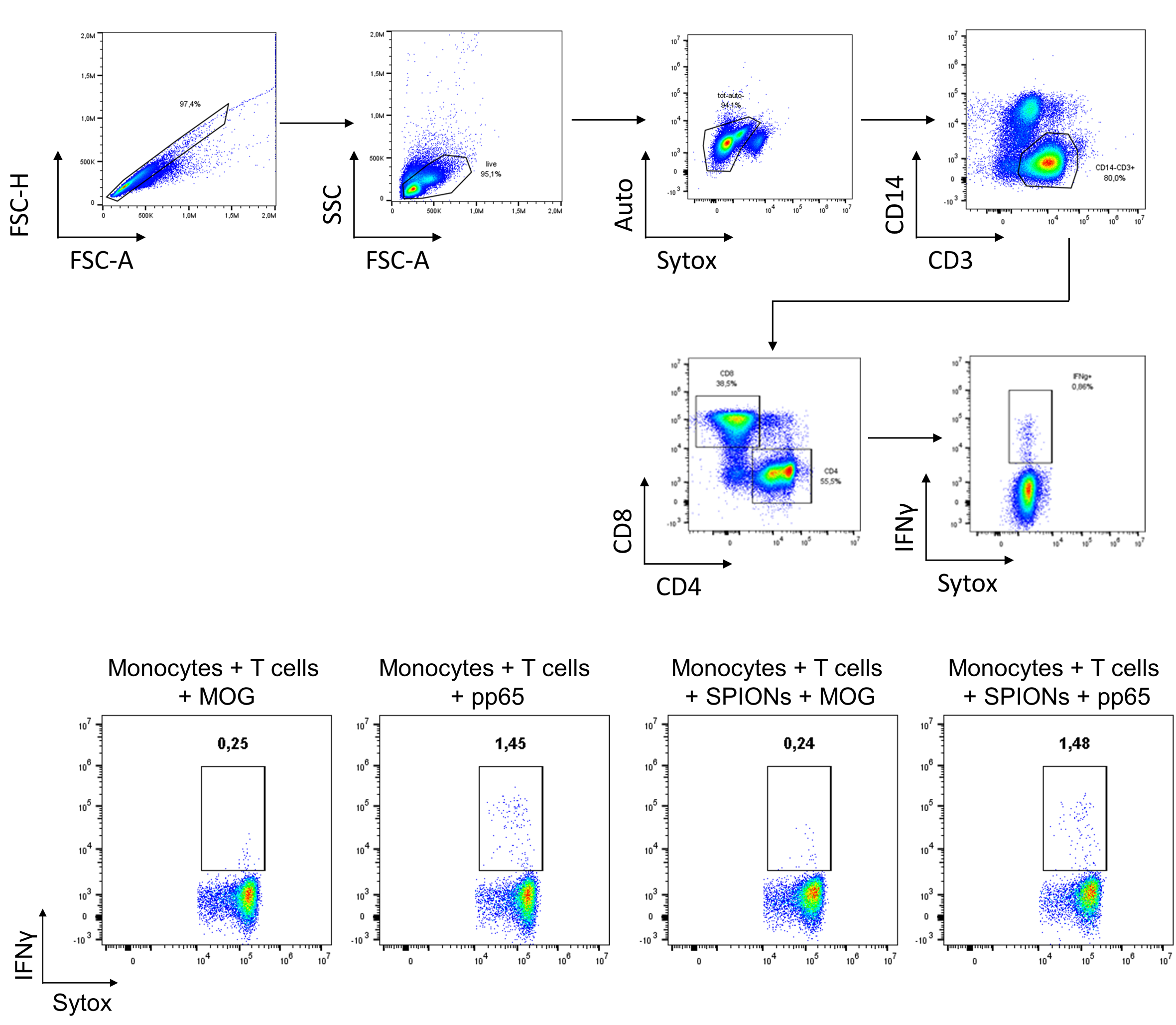

Supplement: Supplementary Figure 8 — Analysis of intracellular IFNγ of CMV-specific CD8+ T cells. (A) Gating strategy. Doublets and cell debris were excluded via forward and sideward scatter, as well as dead cells via SYTOX Blue staining. Auto-fluorescent cells were excluded via a dump channel. IFNγ expressing CD8+ T cells were identified by gating on CD3+CD14-CD8+ cells. (B) Representative blots for mononcytes incubated with unloaded or SPION-loaded T cells in the presence of MOG peptide or pp65 peptide. Sytox, SYTOX Blue; Auto, Auto-fluorescent. [file Image_8.tif]

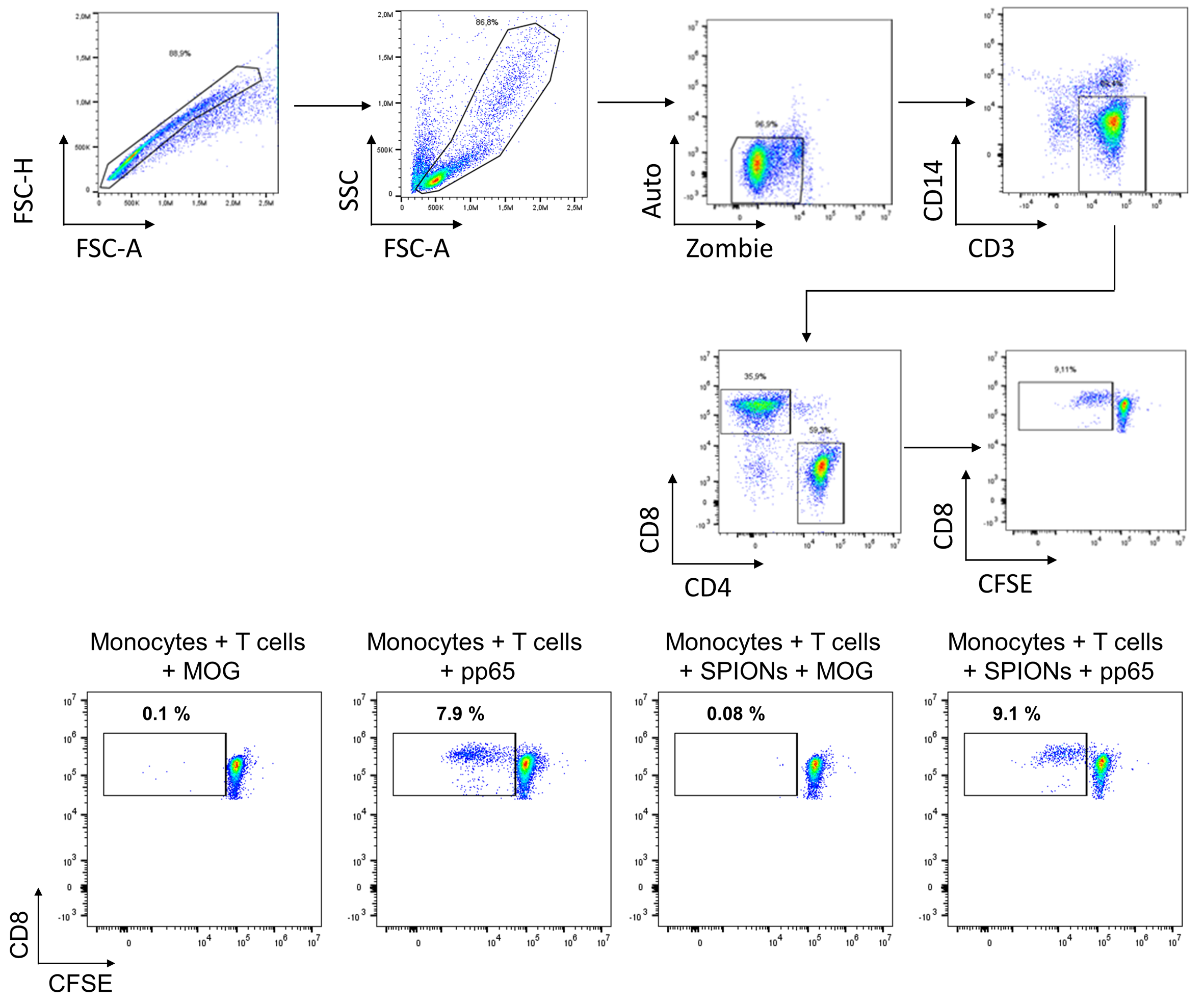

Supplement: Supplementary Figure 9 — Proliferation analysis of CMV-specific CD8+ T cells. (A) Gating strategy. Doublets and cell debris were excluded via forward and sideward scatter, as well as dead cells via Zombie Aqua staining. Auto-fluorescent cells were excluded via a dump channel. Proliferating CD8+ T cells were identified by gating on CD3+CD14-CD8+ CFSE low cells. (B) Representative blots for mononcytes incubated with unloaded or SPION-loaded T cells in the presence of MOG peptide or pp65 peptide. Zombie, Zombie Aqua; Auto, Auto-fluorescent. [file Image_9.tif]
